# Comparison of two combinations of opioid and non-opioid analgesics for acute periradicular abscess: a randomized clinical trial

**DOI:** 10.1590/1678-7757-2016-0407

**Published:** 2017

**Authors:** Manuela Favarin Santini, Ricardo Abreu da Rosa, Maria Beatriz Cardoso Ferreira, Maria Isabel Fischer, Erick Miranda Souza, Marcus Vinícius Reis Só

**Affiliations:** 1 Universidade Federal do Rio Grande do Sul, Departamento de Odontologia Conservadora, Porto Alegre, RS, Brasil; 2 Universidade Federal do Rio Grande do Sul, Instituto de Ciências Básicas da Saúde, Departamento de Farmacologia, Porto Alegre, RS, Brasil; 3 Universidade Federal do Rio Grande do Sul, Departamento de Produção e Controle de Medicamentos, Porto Alegre, RS, Brasil; 4 Universidade Federal do Maranhão, Departamento de Odontologia II, São Luís, MA, Brasil

**Keywords:** Analgesia, Codeine, Periapical abscess, Tramadol, Visual analog scale

## Abstract

Acute periradicular abscess is a condition characterized by the formation and propagation of pus in the periapical tissues and generally associated with debilitating pain.

**Objective:**

The aim of this study was to compare the overall analgesic effectiveness of two combinations of opioid and non-opioid analgesics for acute periradicular abscess.

**Material and Methods:**

This study included 26 patients who sought emergency care in a Brazilian dental school. The patients were randomly divided into two groups: Co/Ac - oral prescription of codeine (30 mg) plus acetaminophen (500 mg), every 4 h, for 3 days or Tr/Ac - oral prescription of tramadol hydrochloride (37.5 mg) plus acetaminophen (500 mg) on the same schedule. Two factors were evaluated: (1) pain scores recorded by the patients in a pain diary 6, 12, 24, 48, and 72 h after treatment, using the Visual Analogue Scale; and (2) the occurrence of adverse effects.

**Results:**

In both groups, there was a reduction in pain scores over time. For the Co/Ac group, there was a significant reduction in the scores 12, 24, 48, and 72 hours after treatment (P<0.05). In the Tr/Ac group, the scores significantly decreased over time from time point 6 h (P<0.05). Comparing the pain at each time point, the groups were not significantly different (P>0.05), i.e., both treatments were effective in controlling pain caused by APA; however, the combination of Tr/Ac caused more adverse reactions as two patients had to stop using the medication.

**Conclusion:**

This study suggests that, considering both analgesic efficacy and safety, the combination of codeine and acetaminophen is more effective to control moderate to severe pain from acute periradicular abscesses.

## Introduction

Acute periradicular abscess (APA) is a condition characterized by the formation and propagation of pus in the periapical tissues as a direct immune-inflammatory result from the root canal infection. Clinical signs include swelling, edema and it is generally associated with debilitating pain^20^ ^,^ ^26^. The clinical approach to reduce pain in APA patients includes the mandatory need to identify the location of the suppurative mass^26^. Initially, pus can be confined to the periodontal ligament space, facilitating a drainage throughout the canal. When pus is located intraosseously, an expansion of the cortical bone (i.e., swelling) is present, which makes patients feel an intense pain. At a final stage, pus breaks the periosteum and invades the submucosal space making the swelling to display a floating aspect with a reduction in pain intensity. At this phase, a surgical drainage of pus is an effective possibility^26^.

In the treatment of APA, besides the local clinical approach, which relies on surgical access and/or root canal debridement and provide pus drainage, a systemic medication oncoming that involves the use of analgesics and antibiotics is also considered. Antibiotics can be prescribed as a complementary measure especially for abscesses associated with systemic involvement, including fever, malaise and lymphadenopathy; disseminating infections resulting in cellulitis, progressive diffuse swelling, and/or trismus; and abscesses in medically compromised patients at increased risk of a secondary infection following bacteremia^1^ ^,^ ^24^ ^,^ ^26^. In APA cases, analgesia becomes challenging because the immunological mechanisms of pain have already been triggered. It is generally accepted the use of non-steroidal anti-inflammatory drugs (NSAIDs) to control the pain associated with inflammatory signs such as swelling. Ahmad, et al.^2^ (1997) found that therapeutic doses of NSAIDs were more effective than the combination of acetaminophen (600-650 mg) and codeine (60 mg) in the treatment of pain caused by the extraction of third molars. However, for pain management of infectious diseases, the use of NSAIDs may mask clinical signs of infection^1^. Therefore, in the presence of infection and pain with moderate to severe intensity, the combined use of oral opioids (such as codeine or tramadol) and non-opioids (such as acetaminophen or aspirin) is considered more appropriate^13^, even though there is a clear lack of clinical evidence to support using these drug combinations in pain controlling, especially in APA cases.

Codeine/acetaminophen is the most frequently used drug combination due to its efficacy and safety for the management of moderate-to-severe acute pain^28^. However, the tramadol/acetaminophen combination has also been proposed as an alternative to control acute pain^22^. The use of tramadol has been proven effective in treating some cases of acute and chronic pain. However, the benefits of its use in the treatment of acute pain of dental origin are not well characterized. Because APA is an infectious process of dental origin that might spawn a severe debilitating pain experience to the patient, it seems relevant to compare the overall effectiveness of the two combinations of opioid/non-opioid medications, as indicated by the analgesic effect and the incidence of adverse reactions. The hypothesis is that no significant difference in analgesic effectiveness is present when combining these two opioids (codeine or tramadol) with acetaminophen (non-opioid) to manage pain in patients with APA.

## Material and Methods

This study was approved by the Ethics Committee of the Federal University of Rio Grande do Sul (12671913.1.0000.5347).

This double-blind, codeine/acetaminophen-controlled parallel design clinical trial was conducted following the Consolidated Standards of Reporting Trials (CONSORT) 2010 guideline. The sample consisted of APA patients who sought emergency care at the dental school of the Federal University of Rio Grande do Sul. Diagnosis was established based on clinical and radiographic findings. Classic symptoms of APA were obtained after subjective expressions reported by the patient.

Sample size was calculated based on the outcome of Mehrvarzfar, et al.^18^ (2012), in which, among other medication, tramadol has been compared to an ibuprofen-based pill. With the aid of OpenEpi 2.3, a two-means based comparison was used with the following input: 95% confidence interval, 80% statistical power and ratio 1. The results indicated the need for 10 patients *per* group in order to observe a significant difference among groups.

### Population

Patients eligible for inclusion in the experiment included 18+ years-old adults diagnosed with APA and reporting spontaneous pain greater than 40 mm as measured in the 0-100 mm Visual Analogue Scale (VAS) (moderate to severe pain)^12^. Patients were excluded if they had taken analgesics or antibiotics within 4 hours prior to emergency surgery, reported allergy to the drugs used in this study, had a history of gastric ulcer, liver or kidney disease, uncontrolled diabetes mellitus or epilepsy, pregnancy or were breast feeding.

### Intervention

All endodontic procedures, performed by the same investigator (M.F.S.) were standardized. Anesthesia was induced with 2% lidocaine with epinephrine 1:100,000. Pulpal chamber was accessed under rubber dam isolation. Next, septic-toxic contents were neutralized with K files #08,10 or 15 (Dentsply Maillefer; Ballaigues, Vaud, Switzerland) and 2.5% sodium hypochlorite (NaOCl) (Farmácia Marcela; Porto Alegre, RS, Brazil). Then, the coronal portions of the canals were enlarged with a LA Axxess drill (Sybroendo; Orange, California, USA) and the work length was electronically determined by the apex locator Apex DSP (Septodont; Paris, Île-de-France, France). The apical part of each root canal was prepared to a size 25 K-file using a crown down technique. The canals were profusely rinsed with 5 mL of 2.5% NaOCl and dried with paper points. A sterilized cotton pellet was placed in the access cavity and subsequently restored with the glass ionomer cement Vitro Fil (Nova DFL; Taquara, RJ, Brazil). Finally, the occlusion was checked.

After the first appointment, the patient signed a consent form outlining the procedure and its possible risks. Subsequently, patients were allocated to one of the following groups: Co/Ac, which received one fixed-dose oral tablet containing codeine/acetaminophen (30 mg/500 mg) every 4 hours for 3 days, or Tr/Ac, which received one fixed-dose oral tablet containing tramadol/acetaminophen (37.5 mg/325 mg) every 4 hours for 3 days. Instructions on how to perform the pain recordings using the VAS were given to each patient. An assistant accompanied the patients while the first dose of medication was taken.

The patients received a pain diary, containing the VAS, and a sealed package with the other doses of medication. If patients experienced pain that was not controlled by the tested medications, they were instructed to take additional medication (acetaminophen 500 mg, every 4 hours) using under an on-demand scheme.

Four days after the first visit, the patient returned the pain diary. To indirectly check adherence to the treatment plan, patients were also asked to return the medication package. Numerical values obtained at each time point (0, 6,12, 24,48 and 72 h after the first dose administration) for each patient were recorded.

In cases that drainage was not possible or in the presence of systemic symptoms (fever, lymphadenopathy, swelling or trismus), 21 amoxicillin capsules (500 mg) were prescribed and given orally every 8 hours for 7 days.

### Randomization and blinding procedures

The random allocation sequence was performed using random table numbers generated at randomizer. org. A pharmacist encapsulated the drugs in identical capsules, and packaged in white bottles numbered 1 to 26. Information on drug administration was described on the label. An assistant who was blinded to the aim and the protocol of the study generated the numbers using a spreadsheet program (Microsoft Excel).

### Outcomes

The primary outcome was the pain scores. They were obtained using VAS before and after the first appointment. Secondary outcomes included frequency of additional medication use and adverse reactions reported by patients. All information was registered in the pain diary by the patient.

The Number Needed to Harm (NNH), i.e., the number of patients receiving Tr/Ac treatment to cause one additional patient to be harmed, compared with patients who received Co/Ac treatment was also calculated.

### Statistical methods

The analyses were performed on an intention-to-treat basis. All statistical analyses were performed using SPSS v.18.0 (IBM Corp.; Armonk, New York, USA).

Descriptive statistics were performed, with presentation of data as absolute and relative frequencies, median and 25^th^ and 75^th^ percentiles.

Student's t-test for independent samples was used to determine differences between the mean ages for each group. The Chi-square test was used to verify differences between the groups regarding gender, tooth type, antibiotic use and frequency of adverse reactions. Mann-Whitney U test was used to identify the difference between the mean initial pain scores between groups.

The two groups were compared considering a score-based evaluation in the same time interval using the Mann-Whitney U procedure. Friedman test was used to compare the results over time within the same group, followed by the multiple comparison test of Friedman when needed. Differences were considered significant at P=0.05

## Results

Table 1 summarizes the baseline characteristics of the two groups. There was no significant difference with respect to gender (P=0.682), age (P=0.350), tooth type (P=0.370), use of antibiotic (P=0.239) or initial pain score (P=0.760).

**Table 1:** Baseline characteristics of the two groups: codeine and acetaminophen combination (Co/Ac) and tramadol and acetaminophen combination (Tr/Ac)

	Co/Pa (n=10)	Tr/Pa (n=10)	P
Characteristic			
Gender			0.628^a^
Male	2 (20%)	4 (40%)	
Female	8 (80%)	6 (60%)	
Age (year)			0.350^b^
Mean ± SD	41.6 ±12.7	45.1 ±16.7	
Minimum - maximum	26 - 67	18 - 68	
Tooth type			0.370^a^
Monorradicular	4 (40%)	7 (70%)	
Polirradicular	6 (60%)	3 (30%)	
Use of antibiotic			0.239^a^
Yes	5 (50%)	7 (70%)	
No	5 (50%)	3 (30%)	
Initial pain score			0.760^c^
Median (P25/ P75)	90.5 (71.2/100)	81.5 (72.2/ 98.5)	

a Chi-square test

b Student's t-test for independent samples

c Mann-Whitney U test

**Figure 1:**
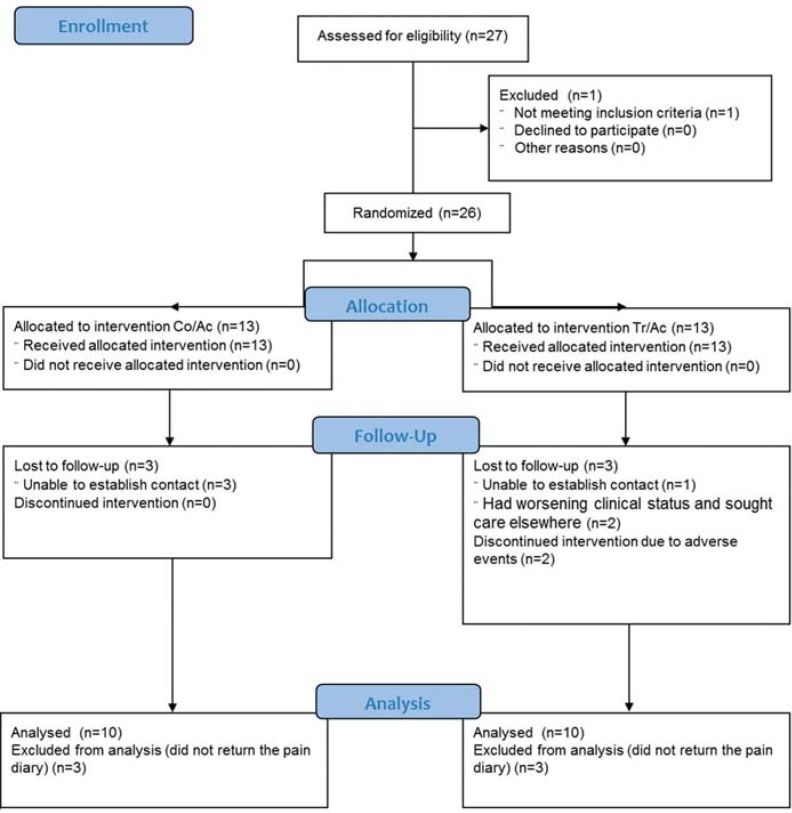
Participants flow diagram

From April 2013 to December 2014, 27 patients were evaluated as potential study participants. Twenty-six patients met the inclusion criteria and agreed to participate in the clinical trial. Out of these 26 patients, three from the Co/Ac group and one from the Tr/Ac group did not return the pain diary and were excluded from the study. Two patients from the Tr/Ac group showed worsening clinical status, sought care elsewhere and also did not return the pain diary. Two patients from the Tr/Ac group dropped out of treatment due to the occurrence of adverse reactions that impaired their daily activities and were excluded from analysis of pain scores, but not from analysis of adverse effects (Table 2). The number of patients in each group, taking into account dropouts and exclusions, is shown in Figure 1.

**Table 2:** Median and 25^th^/75^th^ percentiles of pain scores (mm) before (initial) and after administration of codeine and acetaminophen combination (Co/Ac) and tramadol and acetaminophen combination (Tr/Ac)

	n	Initial	6 h	12 h	24 h	48 h	72 h	P^a^
Co/Ac	10	90.5 (71.25/100)	10.5 (0/ 60.5)	6 (0/ 36.5)	1.5 (0/ 31.7)	0 (0/ 7.7)	0 (0/ 4)	<0.001
Tr/Ac	8	88.5 (74.25/ 99.5)	9.5 (0.5/ 62.25)	5.5 (0/ 62.5)	6 (0/ 39.5)	0.5 (0/ 28)	0 (0/ 20)	<0.001
P^b^		0.733	0.59	0.545	0.676	0.312	0.427	

a Friedman test

b Mann-Whitney U test

**Figure 2:**
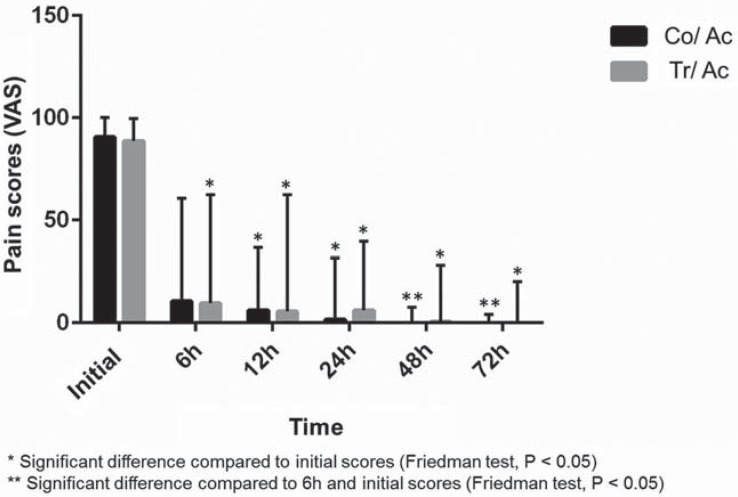
Graph of pain scores at each time point

In both groups, pain scores decreased over time. in the Co/Ac group, pain reduction was significant at 12, 24, 48 and 72 hours compared to baseline (P<0.05). The scores at 48 and 72 hours were also lower than the 6 hours scores (P<0.05). In the Tr/Ac group, pain scores decreased in all experimental time points compared to baseline (P<0.05). The median, 25^th^ and 75^th^ percentile pain scores at each time point are shown in Table 2 and Figure 2.

Forty percent of patients in each group used the additional drug, a number that is not different between the groups (P≈0.999). An average of 1.5 and 1.6 additional tablets per patient was used in the Co/Ac and Tr/Ac groups, respectively.

There was no difference between the groups regarding the frequency of adverse reactions (P≈0.999). Eight patients (80%) from each group experienced at least one adverse reaction, including dizziness, drowsiness, nausea, headache, vomiting and others (Table 3). The NNH for each group was 1.25.

**Table 3:** The frequency of adverse reactions reported during the use of codeine and acetaminophen combination (Co/Ac) and tramadol and acetaminophen combination (Tr/Ac), expressed as the number of cases in relation to the sample size (n)

	Dizziness	Drowsiness	Nausea	Headache	Emesis	Others
Co/Ac	5/10	8/10	6/10	1/10	2/10	1/10
Tr/Ac	4/10	6/10	4/10	1/10	2/10	0/10
P^a^	≈ 0.999	0.628	0.656	≈ 0.999	≈ 0.999	≈ 0.999

a Chi-square test

## Discussion

The combined use of analgesics is indicated to control pain of moderate to severe intensity^22^. Clinical trials have shown that the combination of analgesics with different mechanisms of action can provide greater pain relief than the individual use of each drug^8^ ^,^ ^10^ ^,^ ^16^ ^,^ ^18^.

The maximum daily dosage of paracetamol is 4 grams, bearing in mind that, after the administration of 1 g, the analgesic effect wears off within an average of 6 hours. This dosage must not be exceeded, even if it becomes necessary to reduce the interval between two doses to 4 hours^15^.

In a meta-analysis that used third molar extraction as a pain model^17^, the tramadol/acetaminophen combination (75 mg/650 mg) was superior to the sole use of tramadol or acetaminophen and to placebo with regards to the intensity and duration of analgesia. Using the same pain model, Macleod, et al.^16^ (2002) found that pain intensity was significantly lower after 12 hours in patients who received codeine/ acetaminophen (30 mg/1.0 mg) than reported for patients receiving acetaminophen alone.

In the present study, no difference between the analgesic combinations with respect to pain relief was observed over 72 hours (P>0.05). Both Co/Ac and Tr/Ac treatments were able to decrease the pain scores over time. Therefore, the hypothesis set was partially accepted. In a study by Smith, et al.^27^ (2003) the effects of tramadol/acetaminophen (37.5 mg/325 mg) and codeine/acetaminophen (30 mg/300 mg) in controlling pain after orthopedic and abdominal surgeries in a placebo-controlled randomized clinical trial were similar, as observed herein. In the dental literature, the analgesic combinations studied in the present work were previously compared to other opioids such as hydrocodone with acetaminophen^10^ ^,^ ^11^. A significantly higher pain relief provided by hydrocodone/acetaminophen was observed compared to codeine/acetaminophen (30 mg/300 mg)^10^, whereas a similar effect was found when compared to tramadol/ acetaminophen (75 mg/650 mg)^11^.

The frequency of additional drug use was also not a discriminating factor among groups. In both groups, four patients (40%) took additional acetaminophen. Endodontic literature lacks information about the use of additional drugs in controlling pain of endodontic origin. Some studies in other fields have measured the use of additional drugs as a way to evaluate analgesic efficacy. Da Costa-Araujo, et al.^6^ (2012), for instance, found no differences in the frequency of additional drug use between nimesulide and tramadol treatment groups. On the other hand, Jung, et al.^14^ (2004) found that only patients in the tramadol/acetaminophen group used additional drugs (3%) compared with those receiving codeine/acetaminophen/ibuprofen.

In this study, both groups presented similar frequency of adverse reactions, which is in line with the findings of Smith, et al.^27^ (2003). However, four patients from the Tr/Ac group were lost during follow-up due to an adverse reaction. Two patients (20%) did not tolerate the side effects and two (20%) sought other health service due to the therapeutic inefficacy of drugs tested. Therefore, considering overall safety, the Co/Ac combination could be taken as superior to Tr/Ac group, which partially reject the originally set hypothesis. These data suggest that the safety profile of both associations may be different and future studies must assess this outcome. However, because in this study the sample size calculation was not performed for this outcome (i.e. safety), the result can be a real difference between treatments or a statistic type II error (beta).

Opioid analgesics are useful agents for treating pain of various etiologies, however, adverse effects are potential limitations to their use. In a systematic review, Moore, et al.^21^ (2000) concluded that the addition of 60 mg codeine to acetaminophen produced additional pain relief, but it may be accompanied by side effects, such as drowsiness and dizziness. The parameter used to evaluate the increased risk of an adverse event associated with a drug intervention is the NNH. In this clinical trial, the NNH presented for each group was 1.25, Citrome and Ketter^5^ (2013) affirmed that a NNH in the range of 10-100 may be acceptable for adverse events that may lead to discontinuation, but are not associated with serious immediate health risks. Edwards, et al.^9^ (2002) calculated the NNH for tramadol and tramadol plus acetaminophen in relation to a placebo and found that for single-dose oral tramadol or tramadol plus acetaminophen the NNH for a patient to report any adverse effect were 5.0 and 5.4, respectively. These treatments had high incidences of dizziness, nausea and vomiting. Significantly more nausea, dizziness, and vomiting was reported with single-dose oral tramadol or tramadol plus acetaminophen than with placebo^9^.

Most clinical trials have assessed the management of endodontic pain using a single oral dose scheme^3^ ^,^ ^4^ ^,^ ^18^ ^,^ ^19^ ^,^ ^25^, which allows a more specific evaluation of analgesic efficacy and restricts the assessment of adverse reactions over a limited period of time. In contrast, this study prescribed the tested combinations in fixed-doses, every 4 hours over 3 days. This scheme allows evaluation of the analgesic efficacy and frequency of adverse reactions more closely to a real clinical setting, as well as the analysis of patient compliance. Sadeghein, et al.^25^ (1999) observed that ketorolac (10 mg) was more effective than codeine/acetaminophen (15 mg/325 mg) in controlling pain caused by acute apical periodontitis. Nevertheless, the external validity of these results is questionable because for the baseline pain intensities reported by the authors (greater than 70 mm on VAS), the employed doses of codeine and acetaminophen were low, even when used in combination.

During the phase of abscess development, the pus is aiming to reach the exterior through the bone or periodontal tissue, which explains the pain reported by the patient. Prostration and pain lead the patient to the clinic, however, this situation does not happen and then this clinical condition remains for several days regardless the use of analgesics and antibiotics^7^. In this phase, drainage does not produce much impact, it does not substantially reduce pain and edema. Thus, most patients had postoperative pain and needed analgesics^23^.

It is also important to emphasize that the analysis was performed on an intention-to-treat basis, as referred in the methodology. Our aim was to provide external validity to the study, since in clinical practice analgesics are prescribed to patients developing abscess that use antibiotics or not. Nevertheless, frequency of antibiotics use comparison was performed in both groups, since this could cause confusion when analyzing the analgesic response (Table 1). In homogeneity test, statically significant differences were not observed in regard to the use in the study groups (Chi Square Test, *P*>0.05).

Therefore, the identification of a systemic therapy that presents an overall analgesic effectiveness as measured by its analgesic efficiency and safety is an important clinical subject to improve treatment quality of acute endodonticallyabscessed patients.

## Conclusions

The present randomized clinical trial indicated that Tr/Ac and Co/Ac treatment presented similar analgesic efficacy when used to control pain caused by APA However, Tr/Ac has a worse safety profile than Co/ Ac, resulting in a greater frequency of abandoning treatment due to ineffectiveness or intolerance to adverse effects.
